# Giant Paraesophageal Hernia Presenting With Dyspnea: A Case Report With Surgical Considerations

**DOI:** 10.7759/cureus.81273

**Published:** 2025-03-27

**Authors:** Mauricio Morillo, Álvaro Morillo Cox, Daniella Reinoso Brito, Tatiana Fernandez Trokhimtchouk

**Affiliations:** 1 General Surgery, Hospital de Especialidades Carlos Andrade Marin, Quito, ECU; 2 General Surgery, Clínica Pasteur, Quito, ECU; 3 General Practice, Ministerio de Salud Pública, Quito, ECU; 4 General Surgery, Universidad de las Américas, Quito, ECU

**Keywords:** dyspnea, fundoplication, giant paraesophageal hernia, laparoscopic hiatoplasty, minimally invasive surgery, paraesophageal hernia repair

## Abstract

Giant paraesophageal hernias (PEH) are uncommon and primarily affect older adults. While gastroesophageal reflux disease (GERD) symptoms are the most frequent presentation, some patients develop atypical manifestations, such as dyspnea, due to mechanical compression and impaired diaphragmatic function. We present the case of an elderly patient with a giant type IV PEH who underwent successful laparoscopic hiatal hernia repair with fundoplication. Her postoperative course was uneventful, highlighting the effectiveness of minimally invasive techniques. While surgical repair remains the gold standard for symptomatic and complicated PEH, recurrence remains a concern, necessitating ongoing refinements in surgical strategies. This report reinforces the importance of considering PEH in the differential diagnosis of dyspnea and the role of early recognition and timely surgical intervention in optimizing outcomes.

## Introduction

A hiatal hernia occurs when part of the stomach protrudes through the esophageal hiatus of the diaphragm into the thoracic cavity. These hernias are classified into four types, based on the anatomical structures involved [1-3]. Type I, also known as a sliding hernia, occurs when the gastroesophageal junction (GEJ) migrates above the diaphragm, often leading to gastroesophageal reflux disease (GERD). In contrast, type II, or true paraesophageal hernia, is characterized by herniation of the gastric fundus while the GEJ remains in its normal position. Type III hiatal hernias, which account for over 90% of large hiatal hernias, represent a combination of types I and II, where both the stomach and the GEJ are displaced into the mediastinum. Finally, type IV hiatal hernias occur when the stomach, along with additional intra-abdominal organs, such as the omentum, colon, pancreas, or spleen, herniates into the thoracic cavity. Given their anatomical similarities, types II, III, and IV are collectively referred to as paraesophageal hernias (PEH) [4].

Despite their prevalence, there is no universally accepted definition of a "giant" paraesophageal hernia. Some authors define it as herniation of more than 30% of the stomach, while others set the threshold at more than 50% [4]. This lack of consensus impacts surgical decision-making, but current recommendations suggest that symptomatic PEH should be considered for surgical repair due to the risk of gastric volvulus, incarceration, and ischemia [5].

Most patients with PEH present with symptoms, which may include GERD-related manifestations, such as heartburn, regurgitation, and dysphagia, particularly in type III hernias where the GEJ is displaced into the thorax. Alternatively, patients may present with mechanical symptoms, such as epigastric or chest pain, postprandial fullness, nausea, retching, or dyspnea. In severe cases, gastric volvulus may develop, posing a life-threatening risk [1,5,6].

Understanding risk factors is essential, as they contribute to progressive diaphragmatic weakening and increase the likelihood of symptomatic or complicated herniation. These factors include obesity, chronic increased intra-abdominal pressure (e.g., chronic cough, constipation, or heavy lifting), prior abdominal or thoracic surgical interventions, and connective tissue disorders [7].

The pathophysiology of hiatal hernias involves progressive widening of the esophageal hiatus, weakening of the phrenoesophageal ligament, and alterations in intra-abdominal pressure dynamics. As a result, type IV hiatal hernias may progressively enlarge over time, leading to impaired gastric emptying, chronic compression of adjacent structures, and an increased risk of complications, including gastric volvulus, ischemia, and perforation [8].

We report the case of an 89-year-old female who presented to the clinic with postprandial retrosternal pain and dyspnea. After acute cardiac and pulmonary causes were ruled out, she was referred to the General Surgery Department following a confirmed diagnosis of a type IV paraesophageal hernia. Additionally, this report discusses the varied clinical presentation of PEH and highlights key considerations in its management.

## Case presentation

An 89-year-old female, with a history of arterial hypertension, prior laparoscopic cholecystectomy, hysterectomy, right knee arthroscopy, and right tibia osteosynthesis, presented to her general practitioner with a six-month history of postprandial retrosternal pain, dyspnea, and occasional peripheral cyanosis after food ingestion. The symptoms gradually worsened, prompting her consultation. Her medical history was notable for long-term antihypertensive treatment (valsartan and amlodipine) and a former smoking habit, which she quit 20 years ago.

On admission, the patient’s vital signs were as follows: blood pressure of 130/70 mmHg (normal: 90/60-120/80 mmHg; slightly elevated systolic), heart rate of 70 bpm (normal: 60-100 bpm; normal), respiratory rate of 18 breaths/min (normal: 12-20 breaths/min; normal), temperature of 36.1°C (normal: 36.5-37.5°C; slightly low), and oxygen saturation of 90% on room air (normal: 95-100%; mildly reduced). She weighed 68 kg and measured 1.60 m in height, corresponding to a BMI of 26.56 kg/m² (normal: 18.5-24.9 kg/m²; overweight). Her abdominal and cardiopulmonary examinations were unremarkable, with no signs of respiratory distress or abnormal breath sounds, consistent with the intermittent nature of her postprandial dyspnea.

An initial workup focused on ruling out cardiac and pulmonary causes. A 12-lead electrocardiogram showed no pathologic findings: sinus rhythm, left axis deviation, heart rate of 73 bpm, PQ interval of 190 ms, QRS duration of 90 ms, and no ST-segment abnormalities. Laboratory results were within normal limits, as summarized in Table 1. Additionally, a chest radiograph was performed, which raised suspicion for a hiatal hernia.

**Table 1:** Preoperative laboratory results of an elderly patient with giant paraesophageal hernia.

Parameter	Value	Reference Range
Leukocytes	7200 /µL	4000-11,000 /µL
Neutrophils	66.6%	40-75%
Lymphocytes	26%	20-50%
Hematocrit	48.8%	36-48%
Hemoglobin	16 g/dL	12-16 g/dL
Creatinine	0.82 mg/dL	0.50-1.25 mg/dL
Urea	37 mg/dL	15-45 mg/dL
Glucose	107 mg/dL	70-110 mg/dL
Total Proteins	7.49 g/dL	6.4-8.3 g/dL
Albumin	4.15 g/dL	3.5-5.0 g/dL
Platelets	247,000 /µL	150,000-450,000 /µL
Prothrombin Time (PT)	14.3 sec	11-15 sec
Partial Thromboplastin Time (PTT)	37.6 sec	25-35 sec
Urinalysis	Normal	-

**Figure 1:**
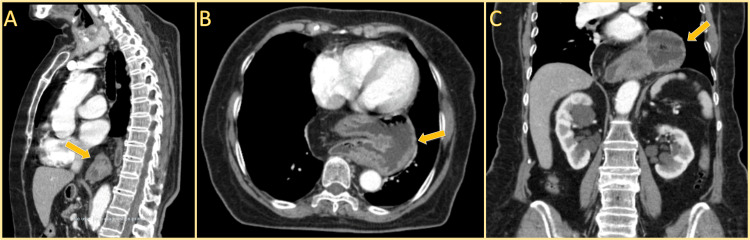
Contrast-enhanced CT scan of the thorax and abdomen demonstrating a giant paraesophageal hernia. (A) Sagittal view showing herniation of the gastric fundus and omentum into the posterior mediastinum (yellow arrow), with displacement of adjacent structures. (B) Axial view illustrating the intrathoracic stomach (yellow arrow) positioned posterior to the heart, consistent with a type IV paraesophageal hernia. (C) Coronal view demonstrating migration of the stomach (yellow arrow) through a widened esophageal hiatus, confirming the presence of a large hiatal hernia.

Subsequently, a contrast-enhanced thoracoabdominal CT scan (Figure 1) was obtained for further characterization. This study revealed a large intrathoracic mass consistent with a giant paraesophageal hernia. Specifically, the scan showed a 12.7 × 6.8 cm herniation of the gastric fundus and omentum into the posterior mediastinum. The esophageal hiatus was widened, and the herniated stomach displaced adjacent structures without evidence of volvulus or strangulation. Additionally, mild diffuse interstitial thickening and scattered ground-glass opacities were noted in both lungs, predominantly at the left basal region, suggesting fibrotic changes. However, the patient had no prior history of pulmonary disease, and these findings had no clinical impact.

Given the progressive nature of her symptoms, the patient was referred to the General Surgery Department for further evaluation. Upper gastrointestinal endoscopy confirmed a hiatal hernia with normal gastric and esophageal mucosa, and esophageal high-resolution manometry showed no motility abnormalities but identified an incompetent lower esophageal sphincter.

Following multidisciplinary evaluation involving general surgery, gastroenterology, anesthesiology, cardiology, and radiology, and given the patient’s symptom burden and diagnostic findings, she was scheduled for elective laparoscopic hiatal hernia repair with cruroplasty and Nissen fundoplication.

**Figure 2:**
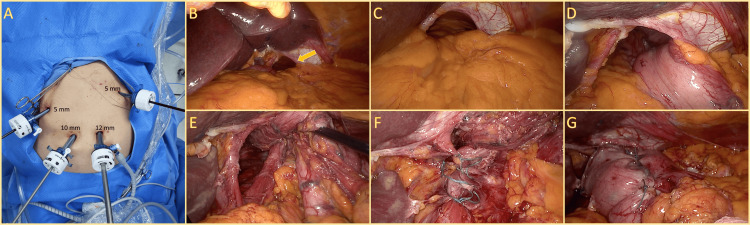
Laparoscopic port placement and intraoperative findings. (A) Trocar placement for minimally invasive approach, showing 5 mm, 10 mm, and 12 mm ports used for access. (B) Initial laparoscopic view revealing a widened hiatal defect (yellow arrow) with the liver retracted for exposure. (C) Close-up of the hiatus, showing the herniated omentum protruding into the thoracic cavity. (D) Reduction of the herniated stomach, returning it to the abdominal cavity. (E) Post-reduction view of the widened hiatus before closure. (F) Hiatoplasty with non-absorbable 2-0 braided sutures approximating the crura. (G) Final result demonstrating a completed Nissen fundoplication, with the wrap securely positioned around the lower esophagus.

Laparoscopic exploration revealed a 4 cm hiatal defect, with 50% of the stomach and omentum herniated into the thoracic cavity. The hernia sac was densely adherent, requiring careful dissection for reduction. After complete mobilization, the gastroesophageal junction was repositioned intra-abdominally, ensuring adequate esophageal length. The hiatal defect was repaired with a non-absorbable braided 2-0 suture, followed by a posterior 360° Nissen fundoplication, which was securely fixed to the right diaphragmatic crus. Figure 2 illustrates the laparoscopic port placement and key intraoperative findings.

The patient had an uneventful recovery, with no signs of respiratory compromise, dysphagia, or reflux symptoms. She tolerated oral intake well and was discharged in stable condition with normal cardiopulmonary function and adequate dietary tolerance.

## Discussion

More than 95% of hiatal hernias are of the type I (sliding) variety, while PEH account for approximately 5%. Among PEH, more than 90% are classified as type III, whereas type II is the least prevalent [4]. In general, PEH are uncommon and primarily affect older adults, with the median age of presentation ranging from 65 to 75 years [9].

The present case illustrates several well-documented clinical features reported in the literature. Although many patients with PEH report GERD-like symptoms, our patient presented with postprandial dyspnea and retrosternal discomfort-symptoms associated with mechanical compression by a large hernia. Dyspnea in particular is underrecognized but has been described in up to 50% of PEH cases, especially in older adults [6]. Additionally, a study of 270 patients undergoing hiatal hernia repair found that 50% experienced early satiety, and 48% reported chest pain [6]. Although the exact incidence of acute complications, such as gastric volvulus or ischemia, remains unclear, these conditions are widely recognized as rare but potentially life-threatening [2,3,10].

In 1986, Cameron et al. described ulcerations at the diaphragmatic hiatus in cases of PEH, where the stomach is pinched, leading to mucosal injury and either occult or overt bleeding. This phenomenon explains the iron deficiency anemia observed in 24-57% of PEH patients [11]. The absence of gastrointestinal bleeding or anemia may reflect the lack of mucosal ischemia or Cameron lesions, consistent with the normal endoscopic findings in this case. CT imaging confirmed a type IV PEH, aligning with current diagnostic standards for atypical presentations [10].

The development of PEH in elderly patients is multifactorial and likely reflects age-related alterations in connective tissue and muscular architecture of the esophageal hiatus. Anatomically, the esophagus is composed of mucosa, submucosa, muscularis propria, and adventitia. The muscularis propria consists of an inner circular and outer longitudinal layer, composed proximally of striated muscle and distally of smooth muscle. A transitional zone exists between these segments. Embedded within the submucosa and particularly at the circular-longitudinal interface of the muscularis propria is a dense network of collagen fibers, which provides tensile strength and elastic recoil essential for esophageal integrity and peristalsis. With advancing age, both the quantity and quality of collagen are diminished, and cross-linking is altered, leading to reduced tensile support and increased susceptibility to anatomical disruption, including widening of the esophageal hiatus [12].

Moreover, aging-related weakening of the phrenoesophageal membrane, combined with repetitive increases in intra-abdominal pressure (e.g., from coughing or constipation), may facilitate gradual herniation of the gastric fundus and other abdominal contents into the mediastinum. While the specific correlation between decreased esophageal collagen content and PEH remains under-investigated, analogous mechanisms are well described in conditions such as achalasia, where abnormal collagen accumulation and smooth muscle remodeling contribute to disease progression [13]. Further studies are warranted to determine whether collagen depletion in the aging esophageal wall or diaphragmatic crura represents a primary etiological factor in giant PEH. This case highlights the need to consider degenerative changes in connective tissue as a plausible contributor to paraesophageal herniation in older adults.

Surgical repair remains the gold standard for symptomatic or complicated PEH, as untreated cases may progress to volvulus, strangulation, or cardiorespiratory compromise. Elective repair in symptomatic patients has been consistently shown to improve symptoms and prevent life-threatening complications [2,14]. Studies have demonstrated that, even in elderly patients, laparoscopic repair provides substantial symptom relief with acceptable perioperative risk [2,15]. As in our case, the decision for surgery was based on progressive dyspnea, postprandial discomfort, and imaging findings consistent with a large type IV PEH, which are accepted indications for operative intervention in accordance with current guidelines [3].

The Society of American Gastrointestinal and Endoscopic Surgeons (SAGES) guidelines recommend the laparoscopic repair as the standard of care, given its efficacy in both elective and urgent cases [3]. Nearly 90% of elective cases can be successfully completed laparoscopically, and approximately 70% of urgent cases can be managed using the same approach [16]. As a result, most trained surgeons prefer laparoscopic PEH repair in both elective and emergency settings. The open abdominal approach is reserved for patients with extensive prior abdominal surgery, whereas the transthoracic approach is considered for those who have failed previous transabdominal repairs. To date, no randomized controlled trials have compared laparoscopic and robotic approaches to PEH repair, and further research is needed to assess the relative benefits of robotic surgery [3].

A successful PEH repair requires complete dissection and removal of the hernia sac from the mediastinum to prevent recurrence, as was performed in this case. The esophagus must be adequately mobilized, ensuring at least 3 cm of intra-abdominal length free of tension. If this cannot be achieved, a Collis gastroplasty is recommended to lengthen the esophagus [17]. Following esophageal mobilization, the hiatal crura are approximated posteriorly and inferiorly to restore the diaphragmatic integrity. The suture technique varies, but most experts recommend interrupted or continuous non-absorbable sutures for a tension-free closure. In large hiatal defects (>5 cm), or cases where primary closure is not feasible, relaxing incisions may be considered. Some authors advocate for reinforcing the closure with biological or absorbable synthetic mesh; however, SAGES guidelines conditionally advise against routine mesh use, as the potential benefits and harms remain closely balanced [3,18].

Recurrence remains a major concern following PEH repair, with reported rates, ranging from 10% to 30%, even with meticulous hiatal closure and fundoplication. While most recurrent hernias are small and asymptomatic, a subset of patients may develop symptomatic reherniation requiring reoperation. Several adjunctive techniques, such as reinforced hiatal closure with mesh and gastropexy, have been proposed to reduce recurrence risk [18].

A recent randomized clinical trial published in JAMA Surgery evaluated the role of anterior gastropexy in PEH repair and found that adding anterior gastropexy reduced one-year recurrence by 21% [19]. The reoperation rate for recurrence was also lower in the gastropexy group (2.5% vs. 8.2%), and the procedure was well tolerated, with only 1.7% of patients requiring suture removal due to pain. These findings suggest that routine anterior gastropexy should be considered as an adjunct to hiatal hernia repair to lower the recurrence risk.

Despite these promising results, long-term outcomes beyond one year remain unclear, and further studies are needed to determine whether gastropexy should be universally adopted or selectively used in high-risk patients (e.g., large defects, prior recurrence, poor diaphragmatic integrity).

Fundoplication is routinely recommended following PEH repair to reduce GERD-related symptoms and recurrence risk. Both complete (Nissen) and partial (Toupet or Dor) fundoplications have been shown to improve postoperative outcomes. However, in patients with preoperative dysphagia or abnormal esophageal motility, a partial fundoplication is preferred. Additionally, wrap fixation in combination with fundoplication has demonstrated favorable outcomes, including a lower recurrence rate, improved symptom control, and reduced risk of complications. The routine placement of pleural or mediastinal drains is not indicated [3,18]. 

Laparoscopic PEH repair is associated with low morbidity and mortality rates. In a series of 662 patients, Luketich et al. reported a 30-day mortality rate of 1.7%, with the most common complications being pleural effusion (9%) and pneumonia (4%). Long-term quality of life scores related to GERD symptoms were rated as good to excellent in 90% of patients. Radiographic recurrence was observed in 15.7%, although only 3.2% required reoperation [9].

This case underscores the importance of considering paraesophageal hernia in the differential diagnosis of dyspnea, particularly in elderly patients. Although GERD-related symptoms are the most common presentation, mechanical complications such as gastric compression, impaired diaphragmatic movement, or intermittent obstruction may also contribute to respiratory symptoms. Timely recognition and appropriate surgical intervention are key to preventing serious complications and improving quality of life.

Surgical repair remains the gold standard for symptomatic and complicated PEH, with laparoscopy offering excellent outcomes in terms of symptom relief, morbidity, and long-term recurrence risk.

## Conclusions

This case underscores the importance of recognizing atypical presentations of giant paraesophageal hernias, such as dyspnea, to avoid delays in diagnosis. Laparoscopic repair with fundoplication remains the gold standard, offering low morbidity and high symptom resolution rates. While recurrence remains a concern, recent advancements, such as anterior gastropexy, may improve long-term outcomes. Future research should focus on optimizing recurrence prevention strategies and evaluating the role of robotic-assisted PEH repair.
